# Clinical Long-Read Sequencing Test for Genetic Disease Diagnosis

**DOI:** 10.1001/jamapediatrics.2025.3320

**Published:** 2025-09-22

**Authors:** Isabelle Thiffault, Emily Farrow, Cassandra Barrett, Meadow Scott, Amy Ross, John C. Means, Warren A. Cheung, Adam F. Johnson, Boryana Koseva, Rebecca McLennan, Elin Grundberg, Chengpeng Bi, Carl Schwendinger-Schreck, Byunggil Yoo, Jeffrey J. Johnston, Florencia Del Viso, Vitoria Paolillo, John Herriges, Lei Zhang, Margaret Gibson, Ana S. A. Cohen, Joe Alaimo, Carol J. Saunders, Tomi Pastinen

**Affiliations:** 1 Department of Pathology and Laboratory Medicine, Children’s Mercy Kansas City, Kansas City, Missouri; 2 Division of Clinical Genetics, Department of Pediatrics, Children’s Mercy Kansas City, Kansas City, Missouri; 3 Genomic Medicine Center, Department of Pediatrics, Children’s Mercy Kansas City, Kansas City, Missouri

## Abstract

This diagnostic study examines the diagnostic yield and turnaround time of long-read sequencing compared to standard-of-care approaches.

Recent work in genome sequencing (GS) shows a 4% to 8% increase in diagnostic yield over exome sequencing (ES) alone, albeit the overall diagnostic gain (<1%) is marginal if GS is compared to combinations of ES with other standard tests.^1^ Recently, we reported undiagnosed cases following GS could be recovered by long-read sequencing (LRS), due in part to the simultaneous detection of clinically relevant repeat expansions,^2,3,4^ methylation abnormalities,^5^ and improved structural variant detection.^6^ However, no study has assessed the implementation of LRS as a first-line clinical test. Herein, we report the comparative diagnostic yield and turnaround time and evaluate the ability to consolidate standard-of-care (SOC) approaches using a single clinical LRS test.

## Methods

Clinical high-fidelity (HiFi) LRS was performed on 235 patients aged 0 to 18 years compared to an age- and phenotype-matched control cohort of 513 patients. Patients selected for the control group underwent SOC inpatient genetic testing, including expedited ES/GS, karyotype, fluorescence in situ hybridization (FISH), chromosomal microarray (CMA), and targeted panels. The eMethods in Supplement 1 provide further details. The Standards for Reporting of Diagnostic Accuracy (STARD) reporting guidelines were followed.

## Results

**Figure 1.**
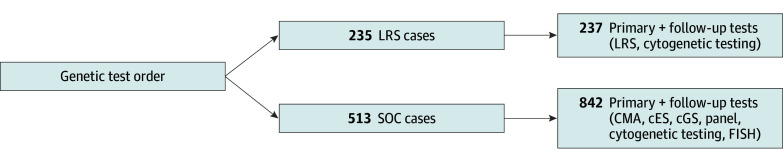
Diagram Reporting Flow of Probands and Number of Test Orders in Long-Read Sequencing (LRS) and Standard-of-Care (SOC) Cohorts cES indicates clinical exome sequencing; cGS, clinical genome sequencing; CMA, chromosomal microarray analysis; FISH, fluorescence in situ hybridization.

LRS case results were compared to controls (Figure 1), with the true diagnostic rate determined by counting diagnoses explaining the primary signs and symptoms. Variants of uncertain significance, carrier status, incidental findings, or pathogenic changes associated with late-onset or variable phenotypes not directly linked to presenting complaint were excluded.

**Figure 2.**
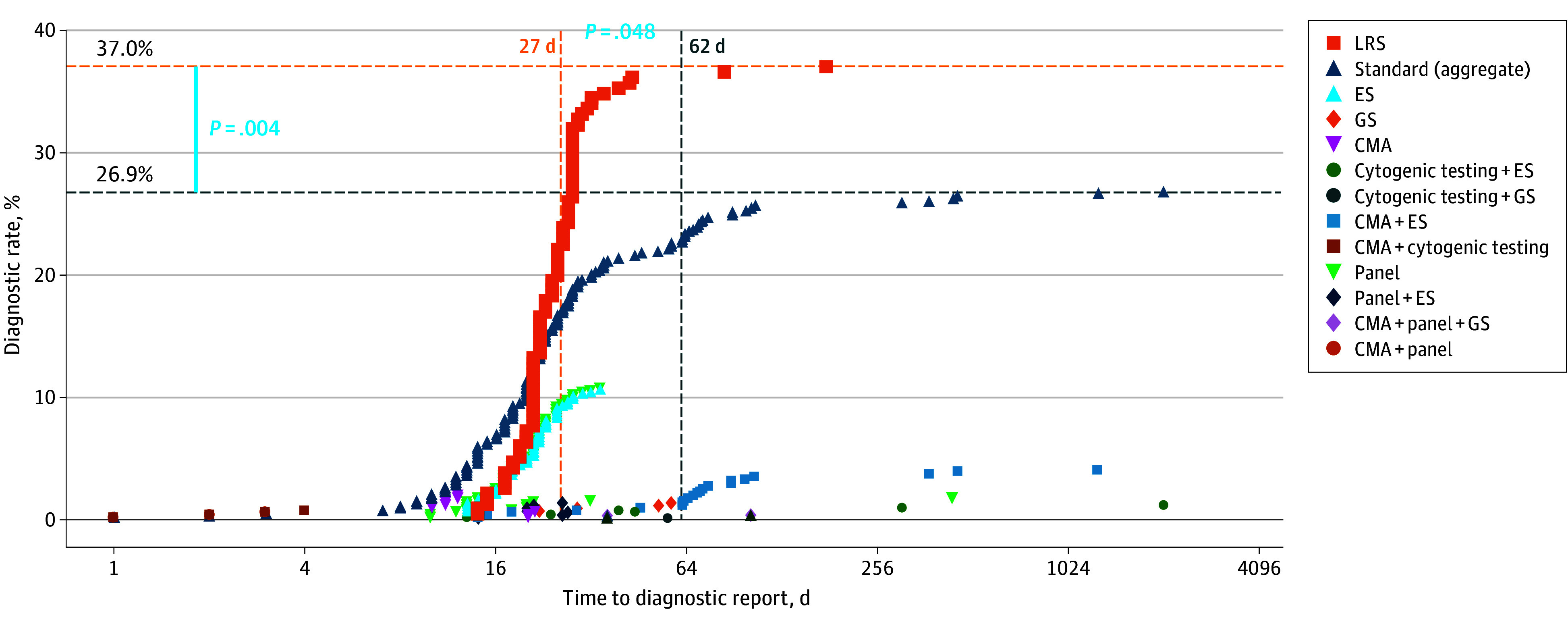
Long-Read Sequencing (LRS) Compared to Standard Genetic Testing Among Matched Pediatric Cases *P* values calculated using Fisher exact test. CMA indicates chromosomal microarray analysis; ES, exome sequencing; GS, genome sequencing.

The LRS cases yielded a significantly higher (*P* = .004, 2-2 χ^2^ test) diagnostic rate compared to the SOC group (Figure 2). The mean length of time from test order to diagnostic report (27 days) was significantly (*P* = .048, *t* test) shorter among LRS cases (Figure 2) compared to controls (62 days). Similarly, if a negative result was returned, the average time to report was significantly (*P* < .001, *t* test) shorter among LRS cases (29 days) compared to controls (91 days).

Factors negatively impacting the time to diagnosis specific to LRS were low sequencing yields requiring reloading and technical limitations of tertiary analysis software. Among the SOC group, the primary cause for delayed diagnosis was multiple test orders required to reach a diagnosis, with an average of 6.1 tests required in the SOC group compared to 2.7 tests in LRS cases. When limited to diagnostic cases, SOC controls had an average of 1.6 test orders for each diagnostic finding (n = 138), compared to 1.01 among LRS cases (n = 87, with 1 diagnosis requiring follow-up cytogenetic testing). If the first test was positive in controls, the mean time to diagnosis was comparable to LRS. However, 57 SOC cases required multiple tests to reach a diagnosis in which the average turnaround time was more than 4 months.

A large fraction of variation in LRS originated from complex structural variants (SVs) and copy number variants, repeat expansions, and methylation variation. Specifically, 16 of 87 reported diagnostic variants in the LRS group (18.3%) benefited from the integrated capability of LRS, which included aberrant methylation, rare expansion disorders, phasing of single-nucleotide variation in a singleton, and detection or refinement of SVs. Note that the availability of standard clinical testing is limited, such as *GNAS* methylation and newly described repeat expansions (eg, in *DIP2B*), which were diagnostic findings captured by LRS. As expected, 71 of 87 diagnostic genotypes (81.6%) without previous standard genetic testing would have been detected by ES, GS, or CMA.

## Discussion

Clinical LRS offers a single, comprehensive genomic assay for diagnosing genetic disease. The advantage of LRS was explained by its expanded variant detection. While the framework used to interpret LRS variation relies on changes also detected by combinations of standard tests, the difference is that in clinical practice, all testing modalities are not typically deployed, and some tests, such as repeat expansions, may not be included in the differential if a presentation is atypical. Due to the sequential nature of SOC testing, there are significant delays in reaching diagnoses compared to LRS. These benefits of clinical LRS are likely only the beginnings of what may be uncovered through expanded testing and further understanding of noncoding regions.^5,6^

## Notes

Supplement 1. eMethods. Supplementary Methods eTable. Characteristics of SOC Testing eFigure 1. Cohorts Used in the Study eFigure 2. Phenotypic Characteristics of HPO Terms Collected for SOC or LRS Testing

Supplement 2. Data Sharing Statement
