# Supplementary material for: Clinical Long-Read Sequencing Test for Genetic Disease Diagnosis
Source: JAMA Pediatr. 2025 Sep 22;179(12):1355–7. doi: 10.1001/jamapediatrics.2025.3320 (PMC12455484; doi:10.1001/jamapediatrics.2025.3320)
Supplement: Supplement 1. — eMethods. Supplementary Methods eTable. Characteristics of SOC Testing eFigure 1. Cohorts Used in the Study eFigure 2. Phenotypic Characteristics of HPO Terms Collected for SOC or LRS Testing [file jamapediatr-e253320-s001.pdf]

Supplemental Online Content

Thiffault I, Farrow E, Barrett C, et al. Clinical long-read sequencing test for genetic disease diagnosis. *JAMA Pediatr*. Published online September 22, 2025. doi:10.1001/jamapediatrics.2025.3320

eMethods. Supplementary Methods
eTable. Characteristics of SOC Testing
eFigure 1. Cohorts Used in the Study
eFigure 2. Phenotypic Characteristics of HPO Terms Collected for SOC or LRS Testing
eReferences.

This supplemental material has been provided by the authors to give readers additional information about their work.

© 2025 Thiffault I et al. *JAMA Pediatrics*.

eMethods. Supplementary Methods

Study cohorts

We utilized clinical HiFi LRS performed in the CGGL from October 6, 2023, to February 6, 2025, on 235 patients ranged in age from 0-18 years predominantly (227/235, 97%) admitted to Children's Mercy Kansas City (i.e. inpatient). These cases were compared to an age and phenotype-matched control cohort of 513 patients selected based on indication for testing by a laboratory genetic counselor not involved in study analysis. Patients selected for the control group underwent SOC inpatient genetic testing including, expedited ES or GS, karyotype, FISH, CMA and targeted panels within the CGGL laboratory (i.e. same analysis team). In order to match by age and phenotype the SOC cohort testing ranged from 2020-2025 (**eTable 1**). Targeted panels ranged from sequencing of specific single genes to panels combining multiple testing modalities such as for infant hypotonia, which includes repeat expansion testing for myotonic dystrophy, and multiple ligation probe assays for spinal muscular atrophy and Prader Willi/Angelman syndrome. Both cases and controls consisted of parent-child trios, duos or singleton cases. Primary signs and symptoms were captured by Human Phenotype Ontology (HPO) terms, along with past diagnostic test results, differential diagnosis or candidate genes, pertinent family history, and availability of biological parents for testing. Inclusion did not require parental samples for either group.

LRS, analysis, and reporting

LRS was performed on genomic DNA isolated from peripheral blood, tissue, cultured cells, and cultured amniocytes. DNA isolation was performed on the Chemagic 360 (Revvity, Waltham, Massachusetts) or manual salting out[1]. Library preparation was completed using either a manual or automated Hamilton NGS Star protocol following manufacturer's recommendations. Briefly, DNA was sheared using the Diagenode Megaruptor (manual; Diagenode, Liege, Belgium) or by the Hamilton NGS Star (automated, Hamilton, Reno, NV) to ~15,000bp. Libraries were prepared with the SMRTbell Express Template Prep Kit 3.0 (100-938-900, Pacific Biosciences, Menlo Park, CA) following manufacturer's protocol[2-7]. Sequencing was completed on Revio instrumentation to a target depth of ≥30X coverage (Pacific Bioscience, Menlo Park, CA). Mapping, variant calling, and genome assembly was

© 2025 Thiffault I et al. *JAMA Pediatrics*.

performed as previously described[2-7]. Variant analysis for single nucleotide (SNV) and copy number variation (CNV) was performed using Via™, which uses a Symptom Association Probability (SAP) score to rank variants for prioritization as previously described[8] (Bionano Genomics, San Diego, CA). Variants were visualized in Via and Integrative Genomics Viewer (IGV, Broad Institute, Cambridge, MA). Variant interpretation was performed using the 2015 American College of Medical Genetics and Genomics (ACMG) Standards and guidelines for interpreting sequence variants[9] and the 2020 ACMG Technical Standards for interpreting and reporting constitutional copy-number variants[10].

Short tandem repeat (STR) expansions and their sequence composition[6] were analyzed using the Tandem Repeat Genotyping Tool (TRGT), Tandem repeat visualizer (TRVZ) and IGV (Broad Institute, Cambridge, MA). Analysis of repeat expansions was limited to the following 52 loci relevant to pediatric disease: *AFF2, ARX, ATN1, ATXN1, ATXN3, ATXN2, ATXN7, ATXN10, ATXN8/ATXN8OS, AR, BEAN1/TK2, CACNA1, C9ORF72, CNBP, CSTB, DAB1, DMPK, EIF4A3, FMR1, FOXL2, FXN, GIPC1, GLS, HOXD13, HOXA13, JPH3, KCNIP4, LRP12, MARCHF6, MUSK, MED12, NOP56, NOTCH2NLC, NUTM2B-AS1, PABPN1, PHOX2B, PPP2R2B, RAPGEF2, RFC1, RUNX2, SAMD12, STARD7, SOX3, TAF1, TBD, TCF4, TNRC6A, TRIO, XYLT1, YEATS2, ZDHHC14, ZIC2.*

Direct methylation was detected using the 5-methyl-Cytosine (mCpG) kinetic embedded algorithm[2,3,6]. Analysis of methylation alterations was restricted to the following conditions: Beckwith-Wiedemann syndrome (BWS) (isolated IC1 hypermethylation, which accounts for <5% of BWS is not directly detectable with GRCh38 and long read sequencing), *FRA12A*-type intellectual developmental disorder, Kagami-Ogata syndrome, Temple syndrome, Prader-Willi/Angelman syndrome, Myotonic Dystrophy 1, Pseudohypoparathyroidism, Type IA, IB, Diabetes mellitus, transient neonatal 1, Silver Russel syndrome 2, Friedrich Ataxia, and Fragile X syndrome.

Variants in 10 genes affected by pseudogenes or low-complexity regions relevant to pediatric disease were assessed using the Paraphase tool[2,11]: *SMN1/SMN2, CYP21A2, CA4A/B, TNXB, IKBKG, F8, CFC1, NCF1, NEB, STRC.*

© 2025 Thiffault I et al. *JAMA Pediatrics*.

Study Design and Comparative Cohort Characteristics

Over a 16-month period, 235 families had clinical LRS completed. These case results were compared to 513 controls that had SOC testing in the past five years matching by age-at-1st-order (**eFigure 1A**). The matched controls were randomly selected based on order indication by laboratory genetic counselors not involved in LRS or SOC analysis. SOC testing included expedited GS or ES sequencing, chromosomal microarray (CMA), targeted panels (sequencing, repeat expansions, methylation) and cytogenetics (Cyto) including FISH or karyotyping. Each LRS case was matched with 2-3 controls resulting in comparable age distribution among cases and controls and identical male/female ratios (**eFigure 1A, B**). Assessment of phenotypic characteristics by comparison of HPO terms collected for GS/ES or LRS testing indicated comparable patient presentations at test order (**eFigure 1C, 2**). The returned results for all LRS and SOC cases were scrutinized and the true diagnostic rate determined by counting diagnoses explaining the primary signs and symptoms at the time of testing. Variants of unknown significance (VUS), carrier status, incidental findings, or pathogenic changes associated with late onset or variable phenotypes not directly linked to presenting complaint were excluded.

© 2025 Thiffault I et al. *JAMA Pediatrics*.

eTable. Characteristics of SOC Testing

**Testing to date**	**Diagnostic**	**Negative**	**Diagnostic rate per test history**	**Overall diagnostic rate to date**	**Proportion of controls with test history**
**ES**	55	138	28%	11%	38%
**ES+CMA**	21	127	14%	4%	29%
**CMA**	10	27	27%	2%	7%
**GS**	7	24	23%	1%	6%
**ES+CMA+Panel**	2	25	7%	0%	5%
**ES+Panel**	10	16	38%	2%	5%
**ES+Cyto**	6	7	46%	1%	3%
**Panel**	9	2	82%	2%	2%
**ES+CMA+Cyto**	6	3	67%	1%	2%
**CMA+Panel+Cyto**	3	2	60%	1%	1%
**CMA+Cyto**	4	1	80%	1%	1%
**CMA+Panel**	4	2	67%	1%	1%
**GS+Cyto**	1	0	100%	0%	0%
**ES+Cyto+Panel**	0	1	0%	0%	0%
**ALL CONTROL TESTS**	**138**	**375**		**26.9%**	

© 2025 Thiffault I et al. *JAMA Pediatrics*.

eFigure 1. Cohorts Used in the Study

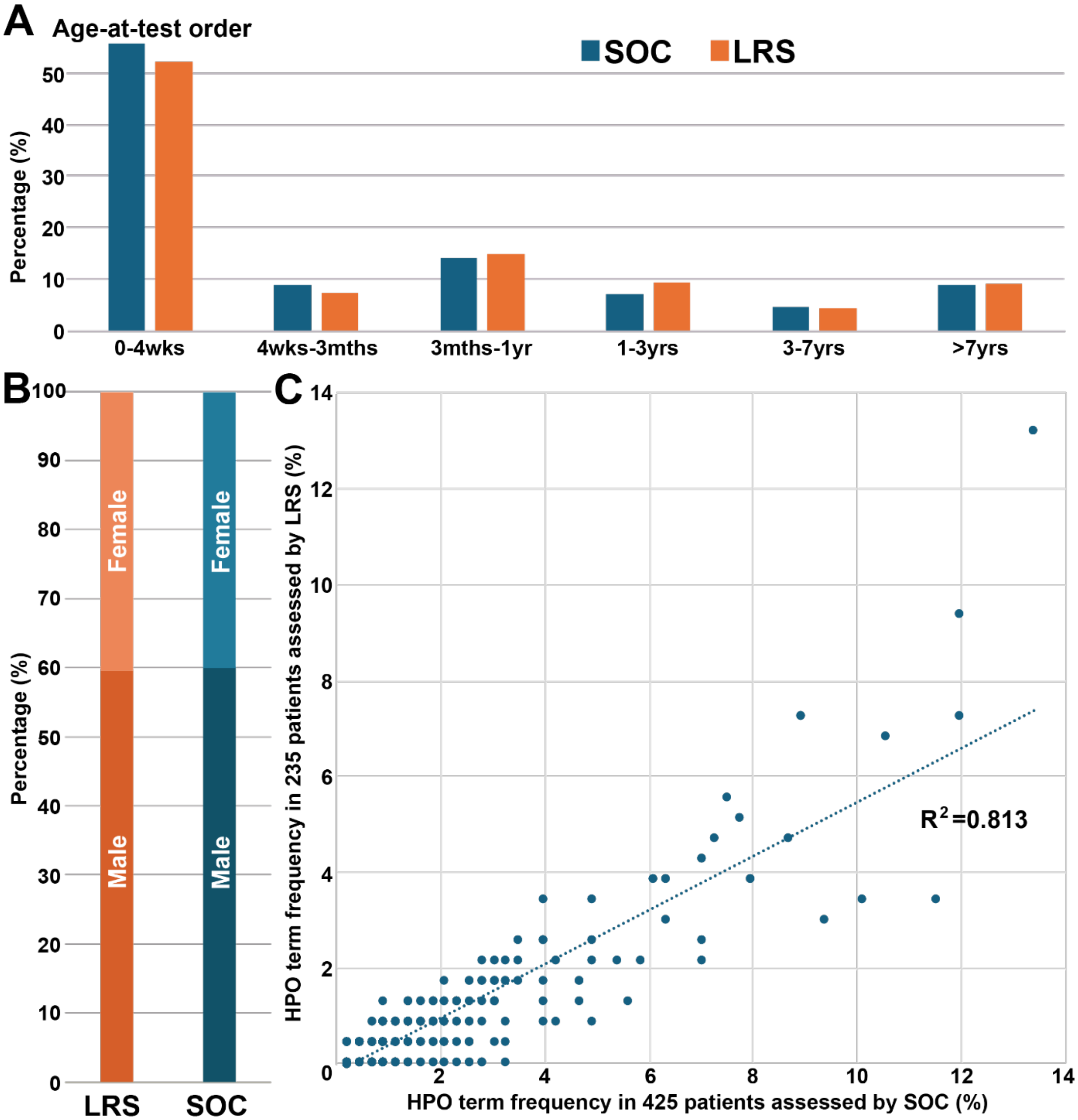

A) Age-at-test order; **B**) Clinical characteristic were highly comparable in LRS and SOC cohorts; Probands were characterized according to sex and the percentage of HPO term frequency; **C)** Comparison of HPO terms collected for LRS and SOC.

© 2025 Thiffault I et al. *JAMA Pediatrics*.

eFigure 2. Phenotypic Characteristics of HPO Terms Collected for SOC or LRS Testing

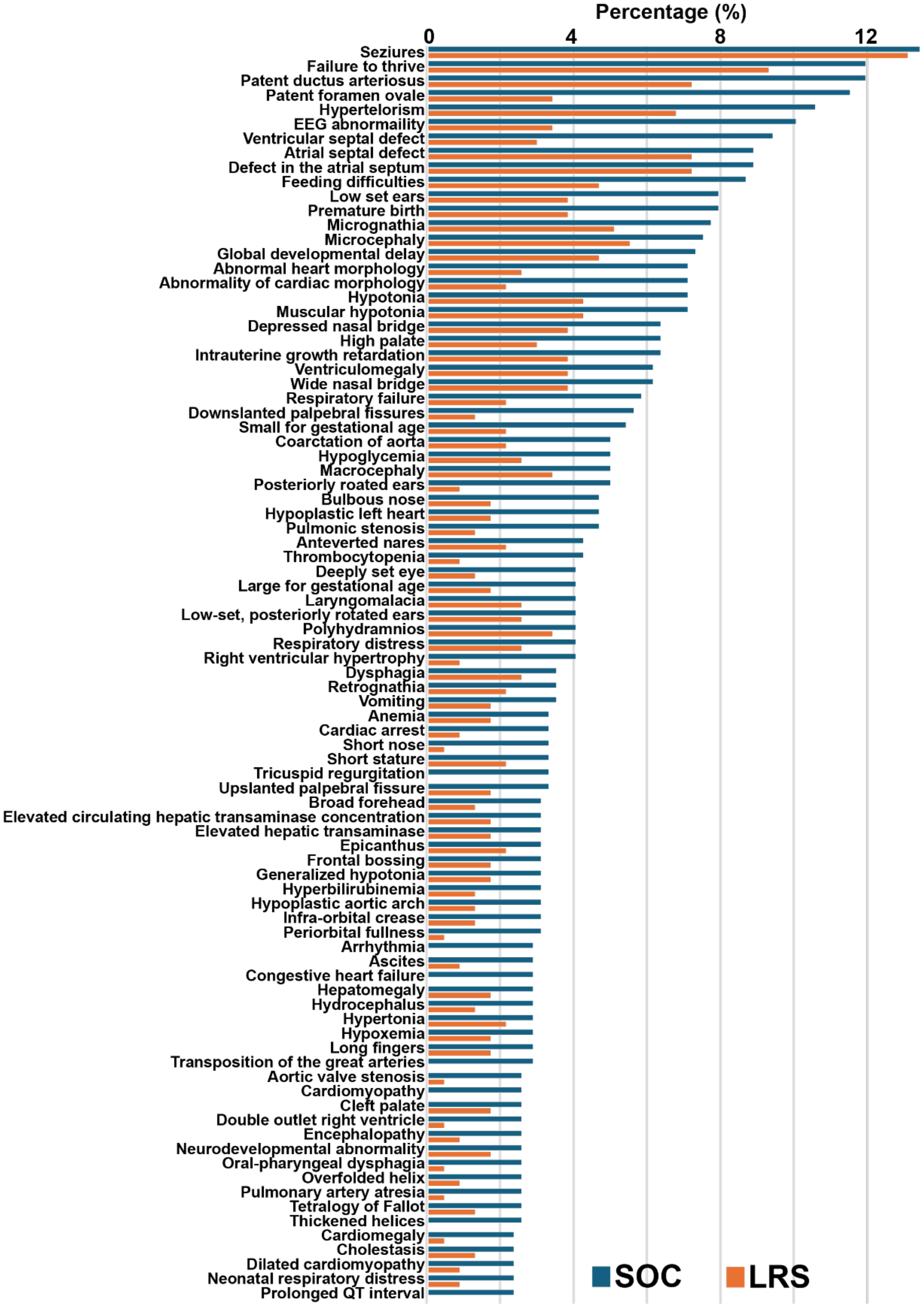

© 2025 Thiffault I et al. *JAMA Pediatrics*.

eReferences.

1. Lahiri DK, Nurnberger JI, Jr. A rapid non-enzymatic method for the preparation of HMW DNA from blood for RFLP studies. *Nucleic Acids Res*. Oct 11 1991;19(19):5444. doi:10.1093/nar/19.19.5444
2. Chen X, Harting J, Farrow E, et al. Comprehensive SMN1 and SMN2 profiling for spinal muscular atrophy analysis using long-read PacBio HiFi sequencing. *Am J Hum Genet*. Feb 2 2023;110(2):240-250. doi:10.1016/j.ajhg.2023.01.001
3. Cheung WA, Johnson AF, Rowell WJ, et al. Direct haplotype-resolved 5-base HiFi sequencing for genome-wide profiling of hypermethylation outliers in a rare disease cohort. *Nat Commun*. May 29 2023;14(1):3090. doi:10.1038/s41467-023-38782-1
4. Cohen ASA, Berrios CD, Zion TN, et al. Genomic Answers for Kids: Toward more equitable access to genomic testing for rare diseases in rural populations. *Am J Hum Genet*. May 2 2024;111(5):825-832. doi:10.1016/j.ajhg.2024.03.016
5. Cohen ASA, Farrow EG, Abdelmoity AT, et al. Genomic answers for children: Dynamic analyses of >1000 pediatric rare disease genomes. *Genet Med*. Jun 2022;24(6):1336-1348. doi:10.1016/j.gim.2022.02.007
6. Dolzhenko E, English A, Dashnow H, et al. Characterization and visualization of tandem repeats at genome scale. *Nat Biotechnol*. Oct 2024;42(10):1606-1614. doi:10.1038/s41587-023-02057-3
7. Farrow E, Jay A, Means J, et al. Case of CLPB deficiency solved by HiFi long read genome sequencing and RNAseq. *Am J Med Genet A*. Dec 2023;191(12):2908-2912. doi:10.1002/ajmg.a.63365
8. Lee B, Nasanovsky L, Shen L, et al. Significance Associated with Phenotype Score Aids in Variant Prioritization for Exome Sequencing Analysis. *J Mol Diagn*. May 2024;26(5):337-348. doi:10.1016/j.jmoldx.2024.01.009
9. Richards S, Aziz N, Bale S, et al. Standards and guidelines for the interpretation of sequence variants: a joint consensus recommendation of the American College of Medical Genetics and Genomics and the Association for Molecular Pathology. *Genet Med*. May 2015;17(5):405-24. doi:10.1038/gim.2015.30
10. Riggs ER, Andersen EF, Cherry AM, et al. Technical standards for the interpretation and reporting of constitutional copy-number variants: a joint consensus recommendation of the American College of Medical Genetics and Genomics (ACMG) and the Clinical Genome Resource (ClinGen). *Genet Med*. Feb 2020;22(2):245-257. doi:10.1038/s41436-019-0686-8
11. Chen X, Baker D, Dolzhenko E, et al. Genome-wide profiling of highly similar paralogous genes using HiFi sequencing. *bioRxiv*. 2024:2024.04.19.590294. doi:10.1101/2024.04.19.590294

© 2025 Thiffault I et al. *JAMA Pediatrics*.
