# Supplementary material for: Clinical Long-Read Sequencing Test for Genetic Disease Diagnosis
Source: JAMA Pediatr. 2025 Sep 22;179(12):1355–7. doi: 10.1001/jamapediatrics.2025.3320 (PMC12455484; doi:10.1001/jamapediatrics.2025.3320)
Supplement: Supplement 2. — Data Sharing Statement [file jamapediatr-e253320-s002.pdf]

Data Sharing Statement

Thiffault. Clinical Long-Read Sequencing Test for Genetic Disease Diagnosis. *JAMA Pediatr*. Published September 22, 2025. doi:10.1001/jamapediatrics.2025.3320

Data
Data available: Yes
Data types: Deidentified participant data
How to access data: https://github.com/ChildrensMercyResearchInstitute/GA4K
When available: With publication

Supporting Documents
Document types: None

Additional Information
Who can access the data: Access inquiries for investigators should be directed to GA4k@cmh.edu (including key to correlate study numbers used in this manuscript).
Types of analyses: For specified purpose
Mechanisms of data availability: After approval
